# Stop treating waste pickers like garbage: An autoethnography on informal waste picking in Brazil

**DOI:** 10.1080/17441692.2023.2201328

**Published:** 2023-04-17

**Authors:** Tara Rava Zolnikov

**Affiliations:** Department of Healthcare Leadership, National University, San Diego, CA, USA

**Keywords:** Waste, recycling, waste pickers, informal industry, occupational health

## Abstract

There are almost 8 billion people on the planet with every single one of them producing some type of waste. The cost of recycling and money made by selling recyclable products has created a strong foundation for informal waste picking to exist. Waste pickers sort through garbage to find recyclable material; it is estimated that there are around 20 million waste pickers worldwide. In 2022, I went to experience life as a waste picker in Brasilia, Brazil for a day to understand issues that may continue to exist in this informal industry. I worked in a triage center and used this experience to inform my autoethnography; even though I have worked in waste picker research for almost a decade, this experience led to a different conclusion. The most interesting issue that I found while working as a waste picker was the lack of uniformity in waste picking, which led to people working on the streets or in co-op triage centers. This population is vulnerable which is worsened from exposure to hazards by nature of the informal environment; this situation contributes to ongoing poor working conditions through lack of governmental oversight, policy development, and change. Ultimately, informality needs to be addressed.

There are almost 8 billion people on the planet with every single one of them producing some type of waste. This scenario has led to unprecedented amounts of garbage in the world. Because of limited land space (especially in urban settings), cost of sorting/recycling/ and managing waste remains a significant issue in the world. While some high-income countries place a financial emphasis on controlling waste through sorting measures (e.g. special bins and trucks for pick up) and recycling centres as well as designated landfill sites, most of the world cannot maintain this high level of management. Recycling is the one aspect of waste management that is profitable, especially if there is a demand for the products in the area (e.g. plastics, metal, etc.) (Hallows & Munnik, 2008).

The cost of recycling and money made by selling recyclable products has created a strong foundation for informal waste picking to exist. Informal waste picking, via individuals or co-ops of waste pickers, sort through garbage to find recyclable material, has a strong presence in the world; it is estimated that there are around 20 million waste pickers worldwide (Workplace Health Without Borders, 2022). In low-income settings, buy-back centres offer money for products thereby creating a value chain (Dilata, 2008; Mueller, 2005; Theron & Visser, 2009; Webster, 2020).


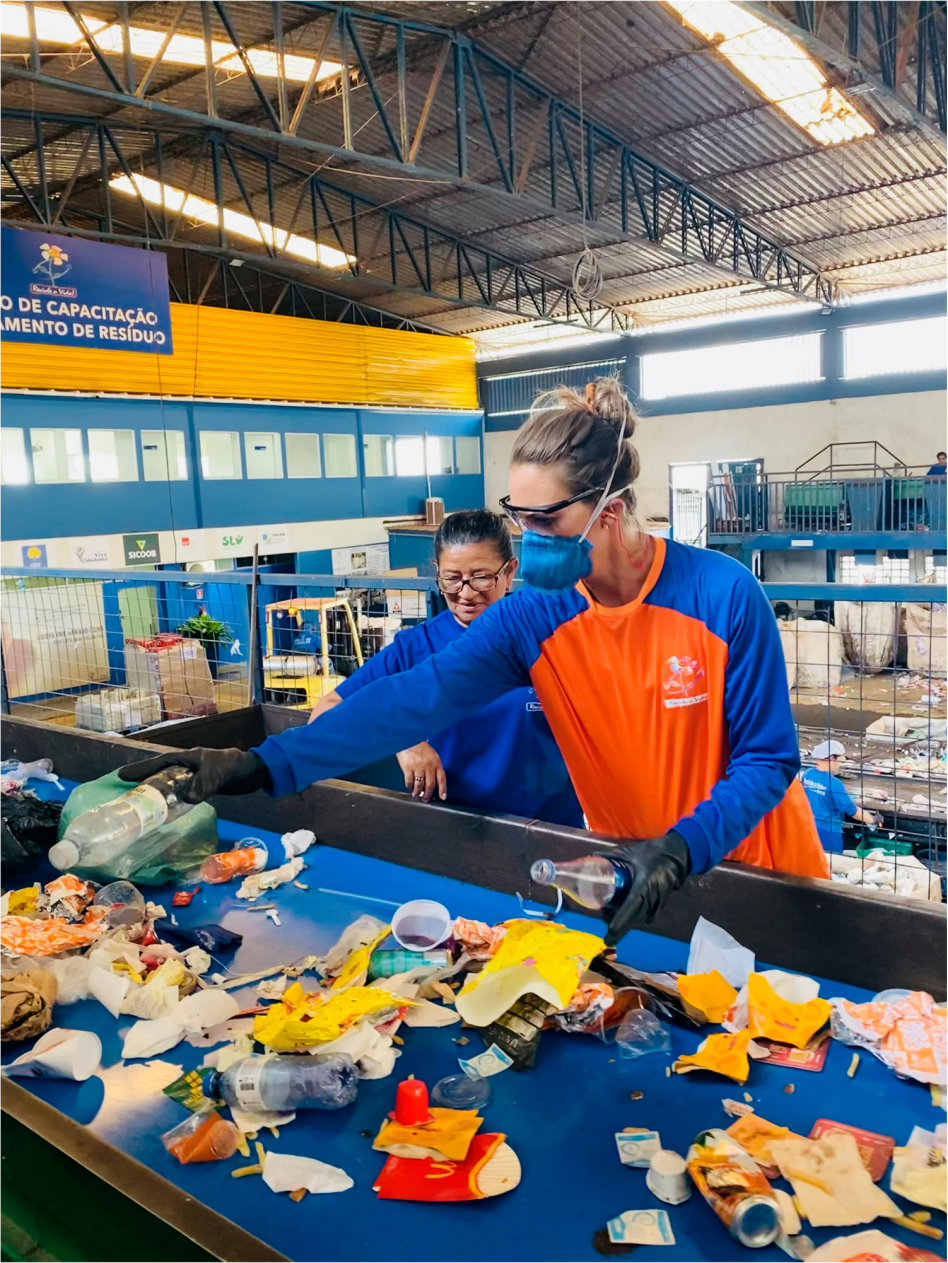
In 2022, I went to experience life as a waste picker in Brasilia, Brazil for a day to understand issues that may continue to exist in this informal industry. The amount of time I spent working with them clearly has its limitations, but besides this snapshot in time, I have worked with waste pickers for nearly a decade understanding their health-related issues; I have been an active qualitative researcher and published and presented a significant body of work on occupational hazards of waste pickers, but have also moved beyond academia and was one of the creators of a working group within Workplace Health Without Borders that includes a worldwide team whom collectively gathers, discusses, and plans policy or educational materials for waste pickers (see: https://whwb.org/waste-workers-occupational-health-and-safety/). I am very familiar with the industry and hazards associated with waste pickers; I have been immersed in hundreds of interviews from waste pickers and have attempted to understand their lives from these stories. That said, I wanted to understand the issue from a different perspective to gain more intimate familiarity of waste pickers practices and perceptions. I employed an autoethnography, which is a type of qualitative research that uses personal experience and self-reflection to provide cultural insight and interpretation. An autoethnography encourages a researcher to move beyond reviewing an issue from the lens of the target population to experiencing, examining, and critiquing the issue via self which can offer a wide-angle lens and broader space for exploration (Holman, Jones, Adams, & Ellis, 2013; Mitchell, 2016). My experience took place in Brasilia, Brazil. The former open-air landfill in Brasilia was home to the second largest open-air dump worldwide, covering over 300 acres of land (Cruvinel, Marques et al., 2019; Cruvinel, Zolnikov et al., 2019; this landfill existed for more than half a century until it was recently closed in 2017 by the government, which siphoned waste pickers from the landfill to other areas (e.g. triage centres). This is a scenario that might be typical if other areas of the world opt to halt open air landfills, which is a likely because of cost of land, environmental contamination, and lost profits from valuable recyclable products.
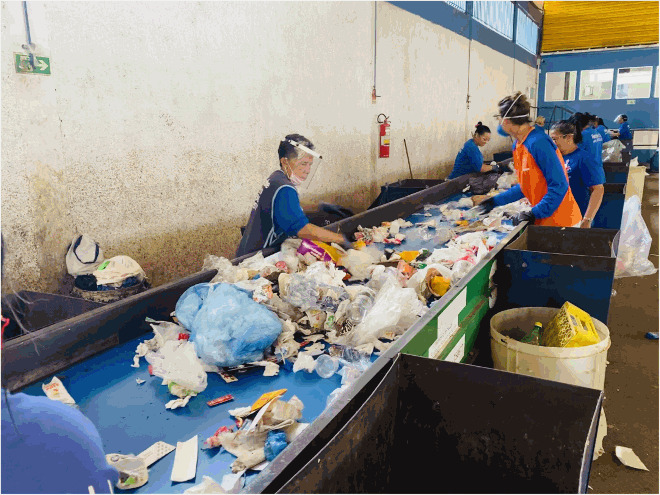


I worked in a triage centre. A triage centre is where recycled materials are sorted from everyday garbage and/or organic materials. There are conveyor belts below where the bags are torn open and dumped for workers to sort through. The triage centre that I worked in was the most organised one that offered the most personal protective equipment; I wanted to experience the opposite side of the scale (from working in the open-air dump) that the waste pickers worked in to see how much improvement was offered. My personal experience was used alongside a field trip which was part of a World Health Organization grant focusing on understanding waste pickers, poverty, vector-borne and other disease exposures. During this time, I was interviewing multiple workers for a phenomenological study (saturation point of n=∼100), so I was fully immersed in an array of waste picker lives and stories. This data is separate from this study but added indirectly to the thick description and data of the autoethnography.

Working as a waste picker in a co-op required concentration. I worked under the pretense that a missed item on the quick-moving conveyor belt would decrease profit, which forced a focus to ensure each piece was being put into the correct bin. The workers on the belt corroborated my story and confirmed that every piece needed to find its correct bin. I oversaw white plastic and clear plastic, which means that every item non-coloured plastic needed to be grabbed and distributed into the correct bin. There were 10 other women on the belt with me, they sorted aluminium cans, paper, coloured plastics, and more. What started off as a mountain of garbage at the beginning of the belt dissipated to nothing after moving through all of the waste pickers hands and sorting bins. I understood the ergonomic pressures which arose from long standing and leaning periods as breaks were few and bar between; I also experienced how tiresome this job could be because of the attention it takes to gather and sort correct materials. The workers also agreed that they did not receive enough breaks and found themselves standing and working for very long periods of time. Literature confirms both adverse occupational health effects in a waste pickers life (Zolnikov et al., 2021).

But the most interesting issue that I found while working as a waste picker in Brazil was not while working in the triage centre – it was the lack of uniformity in waste picking, which I determined as I interviewed waste pickers working in every different type of scenario. On one side of the spectrum is an individual sorting through street garbage or open dumps who exchanges material for money to some type of recycling centre. The other type of informal waste picker works in an organised co-op in a triage centre, which is a large industrial operation that receives mixed or sorted garbage with recyclable material that passes on conveyor belts to workers who separate material into bins. While they are both informal, they are very different from each other. Working on the streets as a waste picker has a barrage of hazards associated with it (e.g. physical, chemical, biological, ergonomic, social, and psychological) and it is estimated that there are still 3000 smaller open-air dumpsites in operation in Brazil (Abrelpe, 2016; Zolnikov et al., 2021). Moving from picking in the streets or open-air dumps has improved life for triage-centre waste pickers by working in groups, having an established schedule, not working outside, and being exposed to less occupational hazards (Zolnikov et al., 2019). However, the individual street waste picker is the most common type in Brazil, which occurs despite government involvement to improve working conditions through triage centres (Zolnikov et al., 2019). That said, the continued amount of individual waste pickers is a separate problem to the major issue that occurred after the closure of the dump and implementation of organised triage centres.

The primary failure of moving waste pickers from streets and open-air dumps to triage centres was that informality of the sector was not addressed. Unfortunately, waste pickers in triage centres continue to suffer from poor work environments (e.g. long working hours, lack of ventilation, etc.), unmet minimum wage rates (e.g. $120/month for workers when the minimum wage in Brazil is $240/month), no private health insurance, and have a lack of labour rights (e.g. occupational hazards that are not protected by labour laws) (Virgeem et al., 2014; Zolnikov et al., 2021). These are all work outcomes that arise and have not been addressed due to informality.

Waste pickers work not by choice, but because of lack of other livelihoods available. This population is vulnerable which is worsened from exposure to hazards by nature of the informal environment; this situation contributes to ongoing poor working conditions through lack of governmental oversight, policy development, and change. Ultimately, informality needs to be addressed. General solutions for change include encouraging private sector, nonprofit organisations, and government branches to prioritise formalisation of waste picking; addressing basic needs of waste pickers to reduce the need to become a street or open-air waste picker; and coordinating research, policy, and practice to shift informality to formality through regulations and ease of formal employment in recycling centres. Brazil initiated change to improve waste picker’s outcomes, like officially closing the world’s second largest open-air dump in Estructural in 2017 and opening co-op triage centres to decrease adverse health outcomes and improve working conditions. There have been positive changes associated with this shift, including a stable salary and continued autonomy in work. But more work is still needed to comprehensively create a sustainable shift in the industry.

As I sorted through garbage, I thought about how I was doing the most important job for the earth today. Despite the utmost crucial nature of waste picking and recycling, working as a waste picker was difficult for so many reasons. This job should continue to be recognised and celebrated, while focusing on addressing informality and ultimately, improving working conditions and health outcomes. Thus, my autoethnography provides evidence of some positive change, though alongside existing gaps that still urgently need to be addressed to uphold international labour standards of freedom, equity, security, and dignity for all working men and women (ILO, 2022).
